# Impact of environmental variables on yield related traits and bioactive compounds of the Persian fenugreek (*Trigonella foenum-graecum* L.) populations

**DOI:** 10.1038/s41598-022-10940-3

**Published:** 2022-05-05

**Authors:** Masoumeh Rajabihashjin, Mehrshad Zeinalabedini, Ali Asghari, Mohammad Reza Ghaffari, Ghasem Hosseini Salekdeh

**Affiliations:** 1 Department of Agronomy and Plant Breeding, Faculty of Agriculture and Natural Resources, University of Mohaghegh Ardabili, Ardabil, Iran; 2 Systems and Synthetic Biology Department, Agricultural Biotechnology Research Institute of Iran (ABRII), Agricultural Research, Education and Extension Organization (AREEO), Karaj, Iran; 3 Department of Molecular Sciences, Macquarie University, North Ryde, NSW Australia

**Keywords:** Ecology, Plant sciences

## Abstract

*Trigonella foenum-graecum* is widely distributed worldwide and grown under a wide range of climatic conditions. The current research was conducted to study the effects of the environmental variables on yield related traits and metabolite contents of 50 different Persian fenugreeks at various geographical locations. Accordingly, multivariate statistical techniques including canonical correspondence analysis (CCA), hierarchical clustering on principal components, and partial least squares regression (PLSR) were applied to determine important proxy variables and establish a relevant model to predict bioactive compounds in fenugreeks. The interrelation of clustered groups emphasized the importance of functional groups of bioactive compounds and several yield related traits. The CCA indicated that two climatic variables of temperature and solar irradiation contributed prominently to 4-hydroxyisoleucine accumulation. The predicted model based on PLSR revealed climatic variables such as temperature, solar, and rain. The precursor of isoleucine was the predictive power for 4-hydroxyisoleucine accumulation while seed weight predicted trigonelline content. The current study's findings may provide helpful information for the breeding strategies of this multipurpose crop.

## Introduction

Fenugreek belongs to the *Trigonella* genus with around 260 species with broad geographical habitats, ranging from the Mediterranean, Asia, Australia, Europe, and Macaronesia to North and South Africa^1^. Thus, Fenugreek is likely to change its chemotype contents, including phenol, flavonoid, alkaloid, protein^2–4^, and its morphometric characteristics for surviving in different ecological conditions. Plant plasticity is an important feature helping the plants adapt to the surrounding situation changes, survive, and produce another generation^5^. It has been well-documented plants’ chemical composition, and morphometric characteristics are more influenced by the environmental conditions and the genetic variation^6,7^. A study showed that the geographical locations had a major role in the variations of the morphometric characteristics among different *Pinus tabulaeformis*populations^8^*.* It has been reported that the chemical composition of the fenugreek genotypes is significantly affected by various environmental variables^9^. Abiotic stresses, such as salinity^10–12^ and drought^13,14^, significantly alter the accumulation of bioactive compounds in Fenugreek under controlled experimental conditions.. Evidence indicates that the content of amino acids and secondary metabolites of different Fenugreek genotypes is modified in different geographical locations like Africa^15^, and Asia^15^. Another report also showed that genotypic variations and environmental conditions mainly control the content of bioactive compounds^16^. Furthermore, there is evidence that different chemical components' abundance is predicted at various environmental conditions in a wild population of *Pilocarpus pennatifolius*^7^. Fenugreek seeds are the most important and well-studied part of the fenugreek plant^17^. However, to date, no study indicates to what extent different environmental variables alter the content of the bioactive compounds with their precursors and yield related traits in the fenugreek seeds in their natural habitats. To this end, the seeds of *T. foenum-graecum* L. were collected from 50 different geographical locations in Iran and their yield related traits were measured using a standard descriptor. Then, the primary metabolites and bioactive compounds of the seeds were measured by the High-performance Liquid Chromatography (HPLC) and Ultraviolet (UV)/Visible (VIS) spectrometry. A series of multivariate statistical analyses were applied to the obtained data for addressing some important issues: (i) to estimate the variations and data grouping as well as to determine the most critical variables in each group; (ii) to understand the relationship and effect of the environmental variables on the fenugreek seeds; (iii) to define the heritability of the plant traits, and finally (iv) to determine the desired prediction model for two bioactive compounds of 4-hydroxyisoleucine and trigonelline and to measure the direct and indirect effects of the traits on these two metabolites.

### Legal statement

The present study complies with relevant institutional, national, and international guidelines and legislation. Seeds of the *Trigonella foenum-graecum* L were collected with permission to collect the seeds of the plant material by the Agricultural Biotechnology Research Institute of Iran (ABRII), project No: ABRII14-05-05-9256.

### Results

#### Analysis of variations

The analysis of variance was conducted to determine the variation in yield related traits and metabolites of the *T. foenum-graecum* L populations (Table 1). The results indicated that 21 traits, such as three environmental variables, seven yield related traits, and 11 metabolites, displayed a significant difference (*P* < 0.001) in the fenugreek’s seeds. Among the environmental variables, the region of Mirash (50.731482°, 36.182436°) was the rainiest location with annual average precipitation of 34.28 mm, while the annual average precipitation in Hamidieh (0.13 mm; 48.516364°, 31.435083°) was almost 280 times lower than Mirash. The mean annual temperature in Hamidieh (35.82°C) was two-fold more than the coldest location in SagzAbad (18.1°C; 49.934878°, 35.766543°). Fasa (53.450804°, 29.137368°) received the greatest amount of annual solar radiation (2211 kJ/m^2^), while Mirash received nearly half of Fasa solar radiation (1692 kJ/m^2^). The bioactive compounds accumulation was significantly different among the locations. 4-hydroxyisoleucine concentration was from 99 mg_/gr dry seed_ in Shushtar (48.832836°, 32.121287°) to 3.36 mg/gr dry seed in Mirash. Trigonelline concentration was from 0.36 mg/gr dry seed in Borujerd (48.813873̊, 33.805145°) to 0.11 mg/gr dry seed in Hamadan (48.527574°, 34.835419°). Furthermore, the maximum value of seed weight was 17.54_gr_ for Kazerun (51.944223°, 29.568908°) in the warm zone at the west-northwest of Iran. In contrast, the minimum (9.54_gr_) rate of seed weight belonged to Khomein (50.317967°, 33.663119°) in the centre of Iran (Table 1).

**Table 1:** Description of 21 measured yield related traits and metabolic traits in fenugreek.

Type	Trait	Maximum	Mean	Minimum	Median
Metabolites	Trigonelline	0.36	0.21	0.11	0.21
	4-hydroxy isoleucine	99.37	46.17	3.36	39.30
	Isoleucine	2.29	0.95	0.16	0.89
	Lysine	0.68	0.39	0.15	0.36
	Phenylalanine	20.38	4.19	1.29	3.93
	Glutamic acid	6.2	1.95	0.22	1.46
	Valine	31.37	18.21	6.72	17.19
	Leucine	17.61	3.37	0.7	2.28
	Aspartic acid	13.75	3.03	0.4	2.28
	Phenol	150.04	111.44	86.2	109.09
	Flavonoid	308.57	217.62	130	227.86
	Antioxidant	0.99	0.31	0.04	0.20
Yield related traits	Seed per pod	16.67	10.53	4.33	10.50
	Seed width	0.43	0.29	0.22	0.28
	Seed length	0.45	0.39	0.33	0.39
	Pod length	16.87	11.64	6.5	11.32
	Pod width	0.5	0.4	0.35	0.39
	Pod number	12.67	6.54	2.33	6.00
	Seed weight	17.54	11.96	9.33	11.63
Environment	Rain	34.28	9.84	0.13	7.69
	Temperature	35.82	25.58	18.77	24.72
	Solar radiation	2217	1957	1692	1940.50

### Genetic advance and heritability of the metabolic traits and yield related traits

The heritability and genetic advance were used to capture the variations explained by the influence of genetics, including the addition, dominance, and epistasis against the environment^18^. The Phenotypic Coefficient Variation (PCV) range was between 4.4 and 76% for phenol and aspartic acid content (Table 2). Furthermore, isoleucine content had the maximum Genotypic Coefficient Variation (GCV) (72%), while phenol showed the minimum rate (3.4%). The inheritance of the total phenol, flavonoid, antioxidant, and trigonelline was medium and equal 60%, 50%, 53%, and 58%, respectively. Furthermore, 4-hydroxyisoleucine had the minimum inheritance (12%). The Genetic Advance (GA) varied from 3.9% for 4-hydroxyisoleucine to 97% for aspartic acid (Table 2).

**Table 2:** PCV, GCV, h^2^, and GA of yield related traits and metabolic traits.

Trait	PCV%	GCV%	h^2^	GA%
Aspartic acid	76	60	0.62	**97**
Glutamic acid	49	61	**0.91**	**91**
4-hydroxyisoleucine	16	6	0.12	3.9
Valine	33	33	**0.90**	61
Phenylalanine	50	68	**0.90**	**92**
Isoleucine	44	58	**0.92**	85
Trigonelline	25	19	0.58	29
Leucine	52	72	**0.90**	**96**
Antioxidant	48	34	0.50	49
Flavonoid	15	11	0.53	16
Phenol	4.4	3.4	0.60	4
Lysine	28	28	**0.91**	52
Pod number	46	45	**0.97**	**91**
100 seed weight	25	31	0.67	34
Pod length	18	18	**0.94**	34
Pod width	32	31	**0.97**	63
Seed length	7.6	6.7	0.77	12
Seed width	9.6	9.1	0.89	17
Seed per pod	26	24	**0.91**	48

The heritability and GA ≥ 90% are shown in bold.

### Hierarchical clustering on principal components (HCPC) analysis

**Figure 1:**
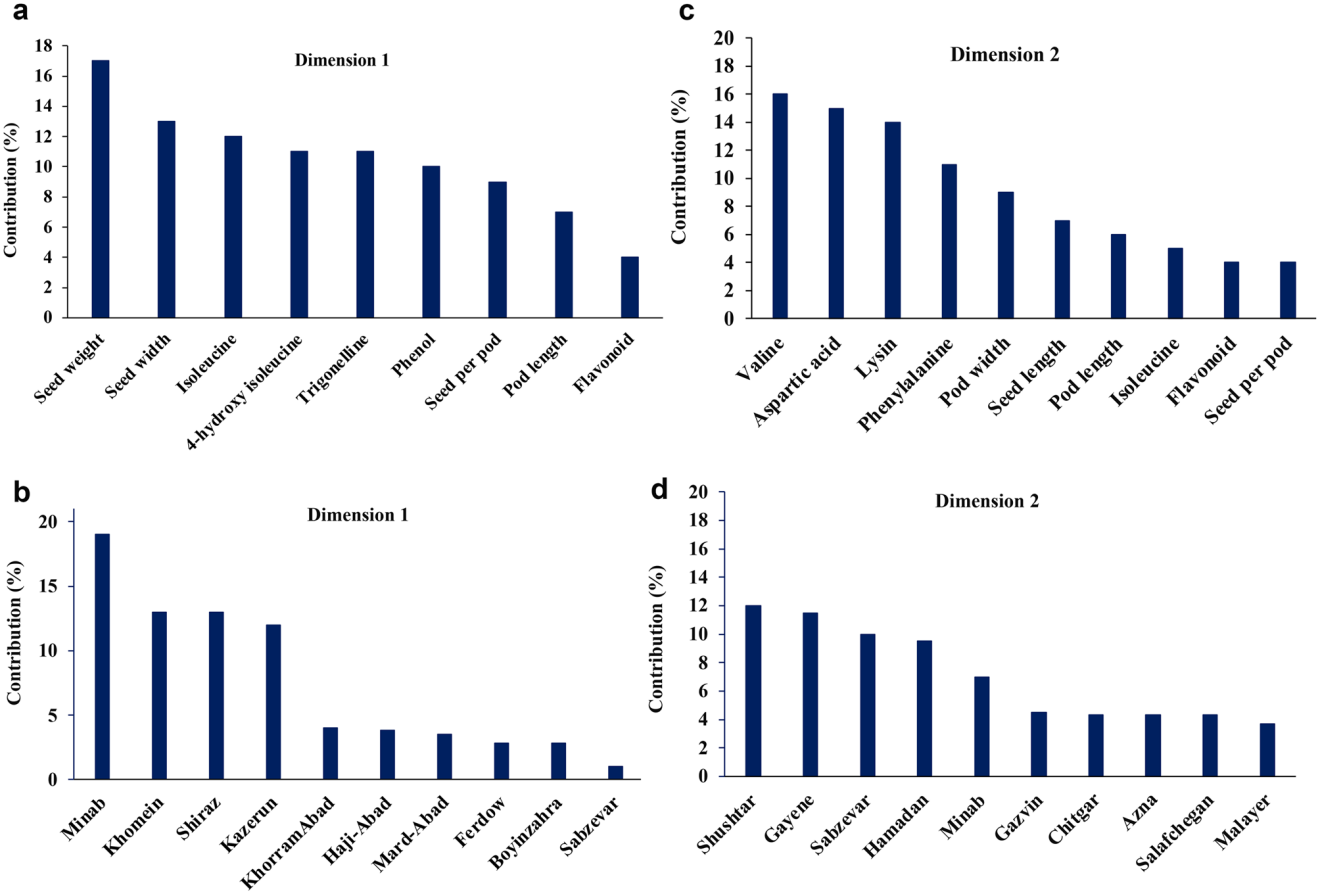
The Bar graph of percentages of contribution yield related triats and metabolite variables a: Minab with 19% and Sabzevar with 0.5% were the highest and lowest contribution variables to dimensions 1 (**a** and **b**), respectively. In dimensions 2 (**c** and **d**), Shushtar with 12% and Malayer with 3% were the highest and the lowest contribution variables, respectively.: seed weight (17%) and flavonoids (4%) showed the highest and the lowest percentage of contribution in dimensions 1 (**a**), respectively. In dimension 2 (**b**), valine (16%) and seed per pod (4%) had the highest and lowest percentages of contribution, respectively.

Hierarchical Clustering on the Principal Components (HCPC) is a convenient method for multidimensional datasets. This method reduces the data dimensionality into less and most crucial information and stable clustering. The results of the Principal Component Analysis (PCA) showed that the two first dimensions explained up 33.6% of the variation among the geographical locations (Figure S1). The PCA loading plot exhibited the two regions of Minab (27.323446°, 56.542827°) and Shushtar (36.182436°, 50.731482°) contributed mostly to dimensions 1 and 2, respectively (Fig. 1). Furthermore, seed weight and seed width had the highest contribution to dimensions 1 (Fig. 1). In contrast, valine and aspartic acid mainly contributed to dimensions 2.

**Figure 2:**
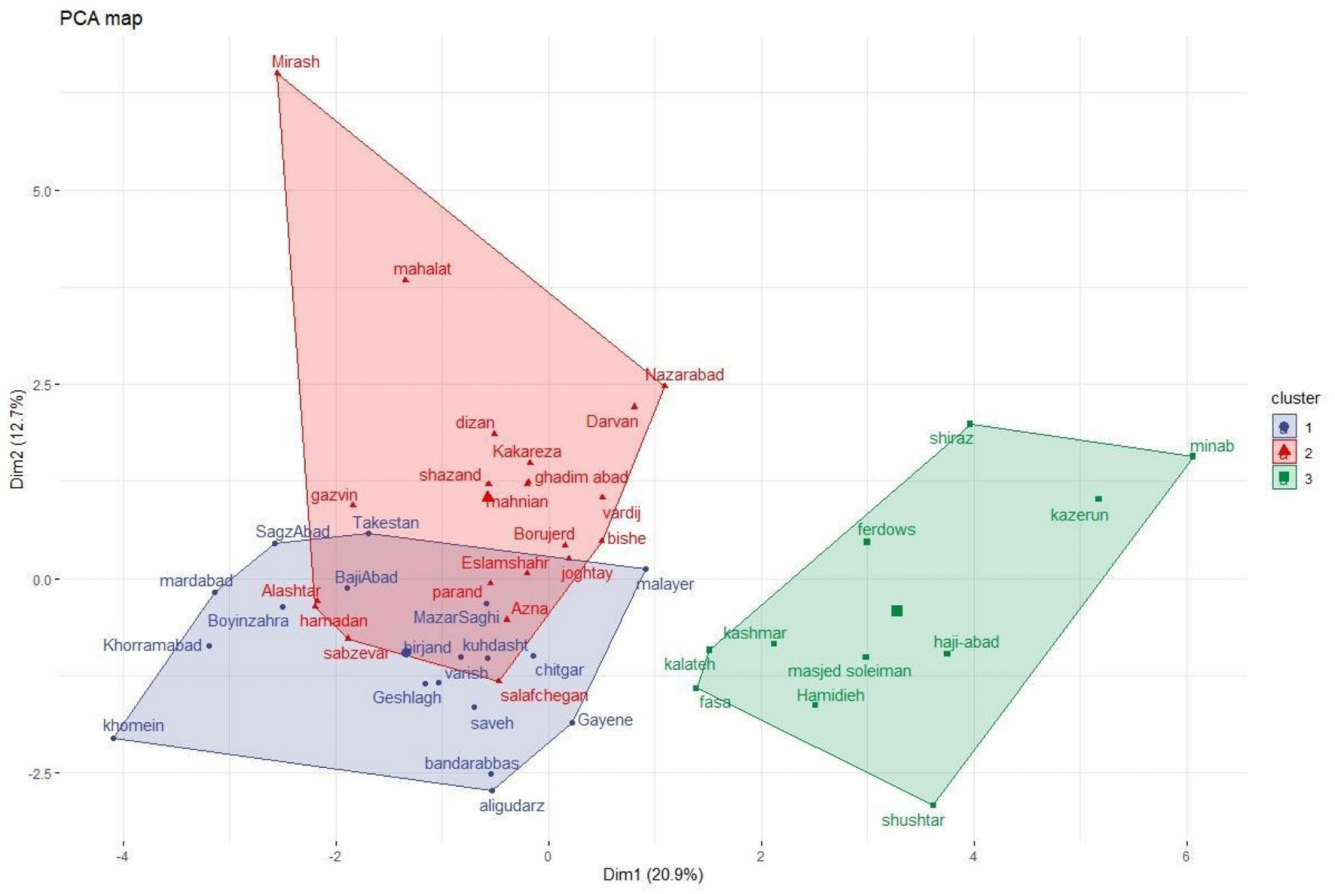
The association among measured variables and two first axis of PCA. The first and second dims explained up 21.4% and 12.9% of the total variation, respectively. The colours of vectors show the contribution rate of each variable to Dim1 and Dim2.

In the second step, hierarchical cluster analysis divided the regions into the three distinct clusters (v. test ≥ 2) (Table 3, Figure S2). The cluster I included 20 members, of which four important variables, including the seed per pod, seed length, aspartic acid, and pod length, passed the significant test (Table 3, Fig. 2, Excel S2, S3). Cluster II had 29 members. The genotypes of clusters I and II were mainly located in the south and the center of Iran and received almost the same average of solar radiation (1954.053 kJ/m2), temperature (25.074°C), and precipitation (9.91 mm) (Excel S2). Cluster III had 11 members and was located in the southwest and northeast of Iran, where the fenugreek’s genotypes receive more light (2047 kJ/m2) and warm (30.23 °C) form of the sun. The average content of metabolites including 4-hydroxy isoleucine (87.86 mg/ gr dry seed), trigonelline (0.25 mg/gr dry seed), isoleucine (1.49 mg/gr dry seed), leucine (4.72 mg/gr dry seed), and flavonoid (230. 86 mgQE/100gr) were higher genotypes located in cluster III than cluster 1 and II.

**Table 3:** Yield related traits and metabolic traits, v. test, and *P*-value, and the most contributed dimensions to clusters I, II and III after HCPC analysis.

Cluster	Variable	v. test	Mean in category	Mean in overall	*P*-value
1	Seed per pod	2.97	12.16	10.52	0.0008
	Seed length	2.72	0.40	0.388	0.0001
	Aspartic acid	2.40	4.17	3.030	0.001
	Pod length	2.02	12.56	11.64	0.002
2	Valine	4.26	23.84	18.21	0.005
	Phenylalanine	3.38	6.28	4.18	0.012
	Lysin	2.44	0.44	0.39	0.027
	Isoleucine	2.17	1.19	0.94	0.03
3	Antioxidant	3.93	0.51	0.30	0.00003
	Flavonoid	3.15	247.25	217.61	0.00005
	Seed weight	3.08	13.273	13.27	0.00008
	Phenol	2.34	119.91	111.43	0.0001
	Trigonelline	2.16	0.24	0.21	0.0007
	4-hydroxy isoleucine	3.93	87.86	46.16	0.0008

We then investigated to what extent the environmental variations exert their impacts on the relationships between traits when the clusters were considered as the three different populations. The correlation between traits of the clusters was investigated by the Pearson correlation coefficient method (Tables S3, S4, and S5). The bioactive compounds of trigonelline and 4-hydroxyisoleucine differed in the direction of correction between the clusters. 4-hydroxyisoleucine was positively correlated with the trigonelline in cluster II. For the traits, 4-hydroxyisoleucine vs isoleucine and lysine vs trigonelline the correlations differed in the direction in cluster I. Seed weight and trigonelline showed the same direction of strong positive correlation in cluster I and cluster III. This correlation is useful for indirect breeding and selection for improved seed yield. The pods' length and seeds number were negatively correlated with isoleucine and 4-hydroxyisoleucine. The paired of traits like phenol, antioxidant, and flavonoid showed the same direction of correlation in all three clusters.

### Canonical correspondence analysis (CCA) between yield related traits and metabolite variables with environmental variables

The Canonical correspondence analysis (CCA) was carried out to identify the link between the metabolites and yield related traits variables with various environmental variables. The result displayed that CCA1 and CCA2 explained 94% of phenotypic variation (Figure S3, S4, and Tables S6, S7). Furthermore, the analysis of CCA also displayed environmental variables can explain the distribution of fenugreeks accessions by affecting metabolites and yield related trait. In CCA1, rainfall was the most significant contribution to environmental variation and positively correlated with the regions with higher average rainfall (17.81 mm) (Excel S3). Furthermore, the most coordinated traits with CCA1 were leucine, seed per pod, and pod length. These traits were positively correlated. However, the correlation between 4-hydroxyisoleucine and trigonelline in the CCA1 axis was significantly negative. In the CCA2, the correlation among temperature, solar radiation, isoleucine, 4-hydroxy isoleucine, trigonelline, phenol, flavonoid, and antioxidant were significantly positive. (Figs. S3, S4, Table S4). Likewise, the average content of isoleucine, 4-hydroxy isoleucine, trigonelline, phenol, flavonoid, and antioxidant in the CCA2 axis was higher than the total mean value (Table S8, Excel S3). Furthermore, the regions which showed a positive correlation in CCA2 had a higher average temperature and solar radiation (27.04̊ and 1980.40 kJ/m2) (Figs. S3, S4, Excel S3) compared with all regions (25.58̊ and 1957.22 kJ/m2).

### Partial least squares regression (PLSR) and modeling

**Figure 3:**
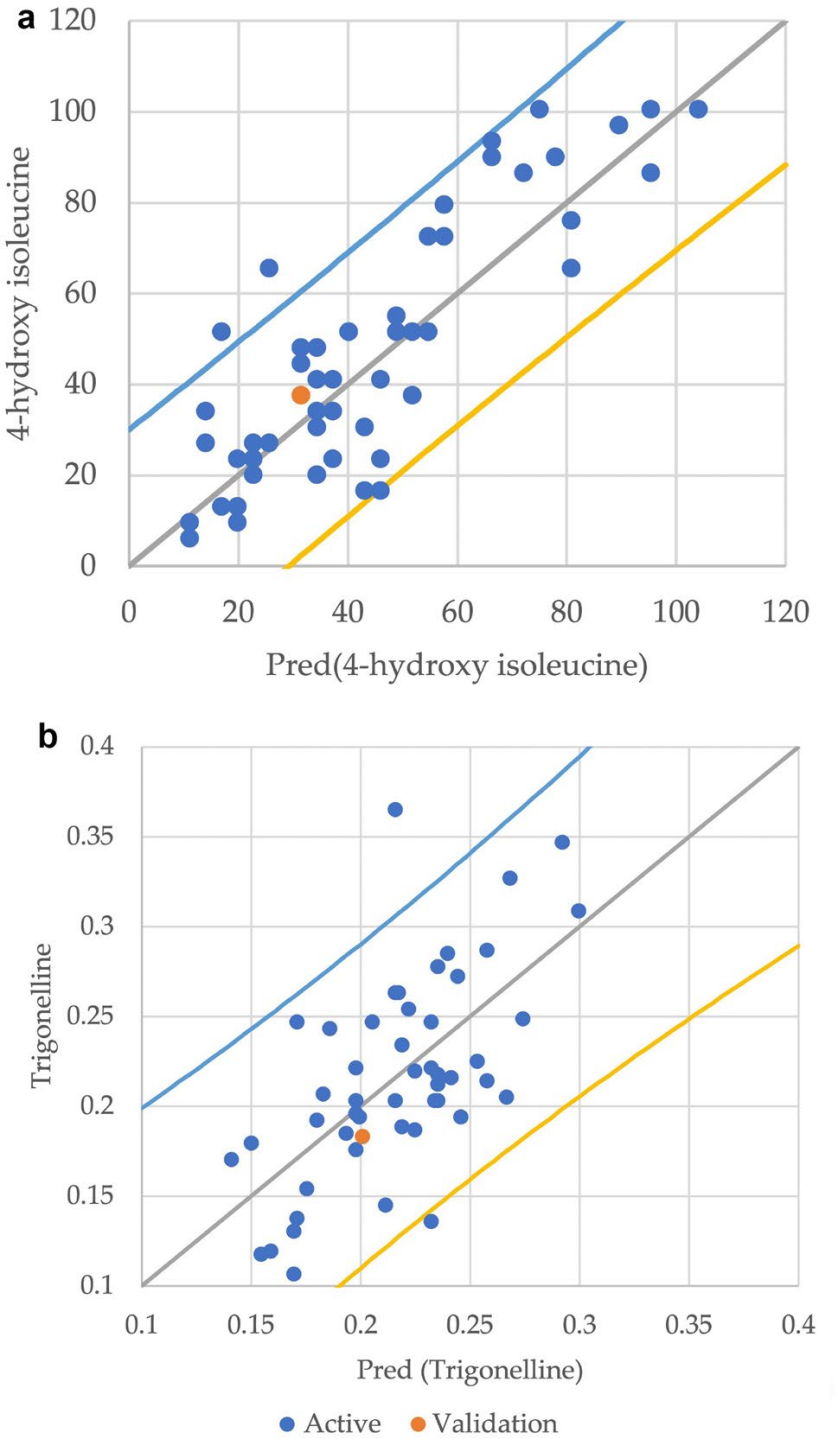
Observed and cross-validated predicted values of 4-hydroxyisoleucine (**a**) and trigonelline (**b**). Each point shows one region. The root mean square error of prediction (RMSEP) indicates the quality of the model in predicting 4-hydroxyisoleucine and trigonelline content. The regression coefficient (R2) indicates the fit between the predicted and observed 4-hydroxyisoleucine and trigonelline content.

Partial least squares regression (PLSR) analysis is a practical statistical analysis for linking the predictors and dependent variable^19^. Since trigonelline and 4-hydroxyisoleucine are the most effective pharmaceutical metabolites in fenugreek^1^, they were chosen as the response variables (Y value). At the same time, the yield related traits, metabolic traits, and environmental variables were considered the latent variables (X values) to find the best predictors for the trigonelline and 4-hydroxyisoleucine yield. In the first step, PLSR was performed by all the variables to determine and eliminate the useless predictors (Fig. 3). Then, the Variable Importance in the Projection (VIP) was assessed for selecting the relevant latent variables^19^. The threshold value for VIP was chosen greater than one (VIP > 1) (Table 4). The results revealed that only seven traits were the most important variables for predicting the 4-hydroxy isoleucine and trigonelline in the model. The seed weight showed the most significant VIP score for 4-hydroxyisoleucine and a lower value for the trigonelline. Environmental variables including the temperature, solar radiation, and precipitation also showed the maximum VIP score value for the 4-hydroxyisoleucine than the trigonelline, confirming that the environmental variables had the greatest effect on 4-hydroxyisoleucine content in the model. However, two yield related traits variables of the seed width and weight showed the highest effect on the trigonelline.

**Table 4:** Validation partial least square regression (PLSR) models for trigonelline and 4-hydroxy isoleucine.

Traits	Trigonelline				4-hydroxyisoleucine			
	R^2^	RMSEP	*P-value*	VIP	R^2^	RMSEP	*P-value*	VIP
Aspartic acid	−0.12	0.052	0.886	0.018	−0.15	22.92	0.644	0.78
Glutamic acid	−0.07	0.050	0.486	0.03	−0.11	22.8	0.785	0.61
Valine	−0.085	0.050	0.903	0.007	−0.14	22.16	0.777	0.70
Phenylalanine	−0.052	0.050	0.304	0.07	−0.067	21.87	0.655	0.24
Isoleucine	0.314	0.048	0.059	0.03	0.32	22.16	0.0008***	**1.74**
Leucine	−0.14	0.047	0.979	0.041	−0.197	22.30	0.939	0.031
Antioxidant	0.087	0.046	0.477	0.87	0.062	23.96	0.0385*	**1.20**
Flavonoid	−0.064	0.044	0.188	**1.04**	−0.066	24.68	0.86	0.31
Phenol	−0.087	0.043	0.288	0.47	−0.104	23.9	0.730	0.32
Lysine	−0.082	0.043	0.439	0.28	−0.112	23.40	0.390	0.33
Pod number	−0.048	0.041	0.723	0.07	−0.083	22.77	0.216	0.41
Seed per pod	−0.009	0.041	0.063.	**1.31**	0.025	21.72	0.261	0.87
Pod length	−0.0511	0.041	0.131	**1.14**	−0.040	20.74	0.536	0.56
Seed weight	0.130	0.040	0.037*	**1.88**	0.220	21.00	0.048*	**1.32**
Pod width	−0.063	0.040	0.195	0.83	−0.066	21.28	0.894.	0.31
Seed length	−0.037	0.040	0.599	0.040	0.080	20.11	0.145	0.75
Seed width	0.061	0.040	0.033*	**1.38**	0.11	20.49	0.078	**1.13**
Solar	0.30	0.040	0.194	**1.32**	0.30	22.38	0.0002***	**1.76**
Temperature	0.26	0.040	0.593	**1.02**	0.23	22.91	0.0006***	**1.77**
Rain	0.146	0.040	0.841	0.93	0.079	22.98	0.0045**	**1.74**

Variables importance in the projection (VIP) greater than one (bold) were used to select relevant latent variables. Root mean square errors (RMSEP) and R^2^ were applied for cross-validation in PLSR analysis.

Significance. codes: 0 ‘***’ 0.001 ‘**’ 0.01 ‘*’ 0.05 ‘.’ 0.1 ‘ ’ 1.

Therefore, the structural equation models are as following for 4-hydroxy isoleucine and Trigonelline:

$$\begin{aligned} & {4-\text{hydroxy}}\;{\text{isoleucine}}\sim - {284}.{917} + {1}.{184}*{\text{seed}}\;{\text{ weight}} + 0.{119}*{\text{solar}}\\ & \quad + {1}.{889}*{\text{temprature}} + 0.{15}0*{\text{rain}} + {12}.{466}*{\text{isoleucine}} + {28}.{983} *{\text{antioxidant}}\\ &{\text{Trigonelline}}\sim - 0.{159} + 0.0{1}0{2}*{\text{seed}}\;{\text{weigth}} + 0.0{56}*{\text{seed}}\;{\text{width}} \\ \end{aligned}$$

## Discussion

In the present study, the effects of the environmental variables were investigated on the yield related traits and bioactive compound accumulation of the Persian fenugreek’s seeds at 50 different geographical habitats. The correlations between the bioactive compounds and yield related traits were also identified. This association might be helpful to exploit the natural diversity and bioengineer the valuable bioactive compound and metabolic pathways in the fenugreek^20^.

### Persian fenugreek showed genetic variability at different geographical locations

Fenugreeks grown in higher temperatures and solar radiation regions had slightly bigger and heavier seeds (Table S8, Figs. S1 & S3) based on CCA and HCPC analyses. This may indicate that the favorable environment for the growth of the fenugreek is the dry and cold temperature at the vegetative stage. In contrast, dry and practically high temperature is favorable for seed production^21^. Plants that grow under high temperatures and solar radiation had higher seed yields^22,23^. The content of phenol and flavonoid in the rainy regions of SagzAbad and Mirash was lower than in drier regions, which is in contrast with the study by Larson result^24^. Warmer regions with higher solar radiation, including Haji-Abad, Kazerun, Kalateh, Fasa, and Bandarabbas, had a higher 4-hydroxyisoleucine, isoleucine, antioxidant, and trigonelline content in seeds. It is proven that phenol content increased under stress conditions such as high temperature and solar radiation to survive plant's life^25,26^. The accumulation of the trigonelline grown under high temperature and sunlight was greater compared to their shade-grown counterparts in the coffee trees^27^. Trigonelline, as an alkaloid^1^, accumulates by the increase in the solar radiation to protect the plant cells against Reactive oxygen species (ROS), leading to the enhanced antioxidant activity^1,28–30^. However, the occurrence of an interrelation between active metabolites and environmental variables does not imply causality. In some cases, the levels of metabolites are dramatically influenced by the stresses or different nutrient regimes^31^.

### Genetic advance and heritability confirmed the effect of the environmental variables on the bioactive compounds and yield related traits

Heritability estimation helps to separate the effect of genetics from nature on the variation in the plants̕ traits^18^. Furthermore, heritability indicates the potential of transmission of the traits from one generation to the next, helping the breeders choose the appropriate selection methods to improve the desired traits. However, broad-sense heritability may not be reliable without estimating the genetic advance^32^. PCV and GCV give us a more distinct picture regarding the influence of the surrounding environment on the heritage of the variables. The traits with high GCV and low PCV are less influenced by the environmental condition^32^. Phenol and antioxidant presented moderately high broad-sense heritability as previously reported^33–35^. In general, phenolic content is mainly responsible for the response to the variation in the surrounding condition of the plants. Although the phenolic compound's inheritance is relatively high, their GA and GCV are too low because of their sensitivity to the environmental condition (Table 2). These metabolic traits, which play an important role in the plant protection and against the oxidative stress in human disease^36^, were related to in the correlation matrix (Tables S3, S4 , S5). Low heritability of the amino acids (Table 2) showed that they are not stable in variations of the environmental condition, as previously reported in the literature^37^.

### Simple and multivariate analysis emphasized functional groups and morphological traits

The environmental variables could predict the quantity of metabolic compounds in the fenugreek. Here, metabolites were measured on samples collected from the field to assess the phenotypic plasticity of fenugreek varieties that showed different magnitudes regarding the correlation between environmental variables and the accumulation of bioactive compounds. It was found that three different patterns of relationships between the yield related traits and metabolic traits in the three clustered populations under different environmental conditions (Fig. 2, Tables S3, S4, S5). Clusters I and II, which have a close geographical location, showed similar correlation patterns (Fig. 2, Tables S3, S5). It was found that the environmental condition influences the correlations between the yield related traits and metabolic traits^38^. The similar correlations among compounds like phenol, flavonoid, and antioxidant in all three clusters may indicate that these compounds are more structurally and functionally related. It has been previously shown that the metabolites with a similar structure were correlated at a global level in the correlation matrix^36,39,40^. There were also significant associations between metabolites in two different sectors of central metabolism, including amino acids and bioactive compounds. Such correlations might be acceptable as the processes related to the biosynthesis of the bioactive compounds rely mainly on the primary metabolite^41^. There was also a relationship between isoleucine as the precursor and 4-hydroxyisoleucine as the end product^42^. A positive correlation between the 4-hydroxyisoleucine and antioxidant activity was seen, suggesting that the antioxidant activity increases upon enhancement of the 4-hydroxyisoleucine content^43^. There were few correlations between any of the yield related traits and metabolic traits. The lower number of such a relationship indicates that although the metabolites are the mediator between the plant's genome and the environment, they are not necessarily the outcome of this interaction. So, it is maybe the reason for the absence of some correlations between metabolites and yield related traits^29^. The heritability of the yield related traits was higher than the metabolic traits (Table 2), which is consistent with the results of several previous reports^41,44–46^. Multivariate data analysis using the PLSR, which is preferable for large numbers and diverse variables, revealed that 4-hydroxyisoleucine was predicted by overlapping sets of the metabolites and yield related traits, indicating their close link to the accumulation of 4-hydroxyisoleucine content in the fenugreek (Fig. 3).

## Conclusion

The current research was examined the effects of the different environmental traits on yield related traits and bioactive compounds on a small panel of 50 different Persian fenugreeks genotypes at different geographical locations. A simple and multivariate statistical analysis showed that metabolic traits had negligible heritability than the yield related traits in the Persian fenugreek accessions. Further, it was shown that the proposed model, predicted 4-hydroxyisoleucine bioactive compound accumulation, is closely related to a set of metabolites and changes in the environmental variables, which in turn will provide helpful information for breeding strategies for fenugreek. However, it is not possible to recommend plant breeding without removing the ecological effect by cultivation under the same environmental conditions. Thus, further information is also needed to confirm that current results are either a plastic response to the environment or genetically controlled.

## Materials and methods

### Plant materials

**Figure 4:**
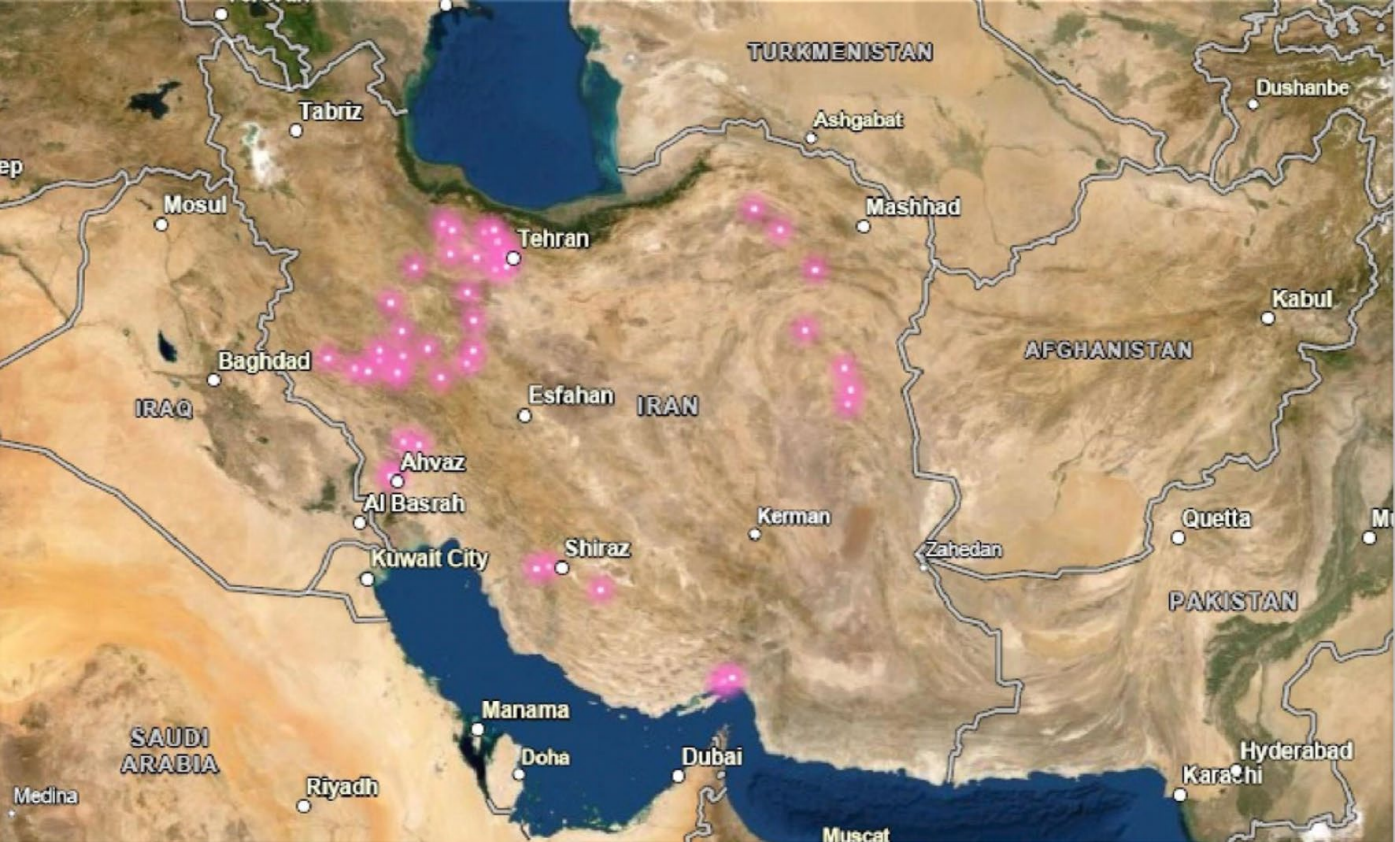
The geographical locations of collected *Trigonella foenum-graecum* L. seeds in Iran. The purple points represent the collected sample’s locations. The map was provided by courtesy of the Earth Science and Remote Sensing Unit, NASA Johnson Space Center (https://eol.jsc.nasa.gov) and was modified using Adobe Photoshop CC v15.2 and Microsoft PowerPoint 2016.

The Fenugreek’s seeds were collected from 50 different geographical locations in Iran (Table S1, Fig. 4). The sampling was done based on standard instruction. Briefly, the fenugreek distribution areas were identified based on the existing flora, such as Iranica, the national herbarium of the Forest and Rangeland Research Institute, and the Ferdowsi Mashhad University. This was chosen based on the difference in the altitude and latitude and the topography of the area, which ultimately causes climate change. Sampling was done randomly with constant distances from each other using the walking distance. The sample unit was landrace. 50 to 100 plants were collected at each site. The collection route was determined using maps at a scale of 1: 500,000. The collection was done at the maximum stage of seeds maturity. In the case of endemic and native crops, the required seeds were obtained directly from the farmers. The data were then entered into a collection form. 100–150 g of each landrace with the completed collection information and forms were sent to the Agricultural Biotechnology Research Institute in Karaj for storage and further evaluation and stored at −20 °C until further analysis. Yield related traits of each landrace and metabolite analysis were carried out on collected seeds at the field.

### Collection of the meteorological data

The climatic data, including average annual precipitation, average temperature, and average solar radiation, were obtained by the *Islamic Republic of Iran Meteorological Organization* (IRIMO). The average Solar radiation (kJ/m^2^) data were obtained from the Global Solar Atlas (http://globalsolaratlas.info).

### Seed yield related traits

Seed features associated parameters with the yield, including the pod number, seed per pod, pod width (mm), pod length (mm), 100-seed weight (mg), seed length (mm), and seed width (mm), were recorded numerically in three replications (Excel S1).

### Measurement of the free amino acids and 4-hydroxyisoleucine

100 mg of the powdered seeds were used to extract the free amino acids and 4-hydroxyisoleucine. Briefly, 1000 ul of 80% ethanol (CAS #: 64-17-5) was added to each tube. Then, the tubes were incubated at 80 °C for 60 min and centrifuged at 10,000 rpm for 5 min. Then, the supernatant was collected and dried in a concentrator^47^. O-phthaldialdehyde (OPA) (CAS #: 643-79-8) was used for the derivatization of the free amino acids. Separation and detection of the amino acids were performed fluorometrically by the HPLC at 340 nm excitation and 450 nm emission (*Knauer*, Berlin, Germany). OPA- derivatives of the amino acid were separated by the Eurospher C18 column (250 mm × 4.6 mm, with five µm of particle size) and incubated at 28 °C. The OPA reagent was a mixture containing 0.05 g of OPA powder, 4500 ul of methanol, and 500 ul of Borate buffer (2.85 g of Borax dissolved in 100 ml of water at 100 °C, pH 9.5) as well as 2 ul of 2-Mercaptoethanol. Solvent A consisted of 80 ml of 0.05 M acetate sodium and 20 ml of methanol. A mixture containing 30 ml of 0.05 M acetate sodium and 70 ml of methanol was used as Solvent B. Furthermore, 100 ul of the sample and 50 ul of OPA reagent were mixed for 30 min before injection. Then, 20 ul of the mixture was used for injection. Chromatographic separations were achieved at a column temperature of 28 °C and a flow rate of 1 mL/min. The ChromGate software was used for peak integration and the data were manually reviewed for quality of integration and compared against a known standard to confirm identities. 4-hydroxyisoleucine was measured fluorometrically at excitation of 330 nm and emission of 440 nm according to the literature^48^.

### Measurement of the Trigonelline

Trigonelline was extracted from 100 mg of the powdered seeds. First, one ml of methanol was added, and the mixture was vortexed for 10 min. Then, the mixture was sonicated (BANDELIN, SONOREX DIGITEC, Germany) for 30 min at room temperature. The supernatants were transferred to the new tubes, and the previous step was repeated as previously described^49^. The quantification of trigonelline was measured using Agilent 1260 Infinity series HPLC (*Agilent Technologies, Santa Clara, CA*) consisting of a No. 616 pump, No. 996 diode array detector as well as No. 717 auto‐sampler, and Aminex column. The mobile phase was water (pH 3.5): methanol (70.0:30.0 v/v) with a flow rate of 0.5 ml/min with an elution time of 6 min. The injection volume was 10.0 µl of the methanolic extract. The absorption wavelength was 263 nm. The OpenLab software was applied for data acquisition.

### Determination of phenol contents

100 mg of the powdered seeds were sonicated in 80% methanol (CAS #: 67-56-1) for 1 h at room temperature and then centrifuged at 5000 rpm for 15 min. The supernatants were collected and transferred to the new tubes, and the volume reached 100 ml by 80% methanol. The phenol content was measured according to the method described by Agbor and Vinson^49^. Briefly, 100 ul of methanolic extract was diluted with 9 ml of water and mixed with 1 ml of Folin-Ciocalteu reagent shaken for 10 min. Thereafter, 10 ml of w/v sodium carbonate aqueous solution was added to the tubes and vortexed for 30 min. The absorbance was measured at 760 nm using the UV–Visible spectrophotometer (*CARY* 330 UV–Vis system) against a blank containing Methanol without extract. The results were expressed as mgQE/100 gr of dry seed.

### Determination of flavonoid contents

The flavonoid contents were measured as previously described by Kim et al.^50^. An aliquot of 1 mL of extract solution, 4 ml of distilled water, and 300 ul of 0.3% NaNO_2_ were vortexed for 5 min. After that, 300 ul of 10% AlCl_3_ and 200 ul of 1 M NaOH were added to the mixture. In the final step, 2.4 ml of distilled water was added to the mixture and thoroughly shaken at room temperature. The flavonoid content was measured at 510 nm against the blank by the UV/VIS Spectrometer (*CARY* 330 UV–VIS system). Quercetin compound was used to quantify the samples and the results were reported as mgQE/100 gr of dry seed.

### Measurement of the antioxidant activity

The Radical Scavenging Activity (RSA) of the crude extract solution of the fenugreek’s seed was used to measure the antioxidant activity using the DPHH (2,2-diphenyl-1-picrylhydrazyl free radical) solution using a method previously described by Molyneux^51^. Briefly, 200 μl of the crude extract was added to 300 μl of methanol and 2400 μl of DPPH solution (0.025 g/l), and vigorously shaken and then incubated in a dark room for 30 min. The absorbance of the mixture was measured at 517 nm using the UV/VIS Spectrometer (*CARY* 330 UV–VIS system). The control had all reagents except seed extract. The percentage of antioxidant activity was estimated using the equation:

1 $${\text{Antioxidant}}\;{\text{activity}}\% \, = absorbance\;of\; \left( {control - extract} \right)/absorbane \;of\; control$$

Control = initial absorbance.

The following formula was used to report the quantification of all primary and secondary metabolites in mg per gr dry seed (Table S2): X (mg/gr dry seed) = (VC)/W, where X = amount of amino acids compounds in the extract, V = final content of the extract solution in ml, W = weight of the extract and C = concentration of standard compounds obtained from the calibration curve.

### Simple and multivariate statistical analyses

Pearson correlation was done by *corrplot*^52^*.* The HCPC method was employed for the PCA and HCA analyses^53^. The GCV and PCV were estimated using the method described by Hanson et al.^54^. Genetic Advance (GA) was calculated by a method described by Herbert et al.^55^. Canonical correspondence analysis (CCA) was carried out by the *vegan*^56^ package of R^57^. Partial least square regression (PLSR) analysis was performed using XLSTAT software. The method of leave–one–out cross-validation was utilized to determine the significant component numbers and Root Mean Square Error of Prediction (RMSEP) for the cross-validation process was used in the PLSR analysis to prevent the overfitting.

## Supplementary Information

Supplementary Information 1.

Supplementary Information 2.

Supplementary Information 3.

Supplementary Information 4.

## Data Availability

All data generated or analysed during this study are included in this published article and its supplementary information files.
