# Supplementary material for: Impact of environmental variables on yield related traits and bioactive compounds of the Persian fenugreek (Trigonella foenum-graecum L.) populations
Source: Sci Rep. 2022 May 5;12:7359. doi: 10.1038/s41598-022-10940-3 (PMC9072307; doi:10.1038/s41598-022-10940-3)
Supplement: Supplementary file 4 — Supplementary Information 4. [file 41598_2022_10940_MOESM4_ESM.pdf]

Supplementary Materials:

Table S1: List of *Trigonella foenume* L. include their locations, longitudes, and latitudes at 50 regions.

Table S2: Equation of calibration curve and R2 value of measured metabolic traits by HPLC and spectrophotometer.

Table S3: Correlation matrix between 19 morphometric ant metabolic variables in fenugreek using Pearson's correlation coefficients (r) for cluster I. Significance P-values: P-value (0 '***' ,0.001 '**' ,0.01 '*') are displayed by dark, medium, and light shading of blue colour.

Table S4. Correlation matrix between 19 morphometric ant metabolic variables in fenugreek using Pearson's correlation coefficients (r) for cluster II. Significance P-values: P-value (0 '***' ,0.001 '**' ,0.01 '*') are displayed by dark, medium, and light shading of blue colour.

Table S5. Correlation matrix between 19 morphometric ant metabolic variables in fenugreek using Pearson's correlation coefficients (r) for cluster III. Significance P-values: P-value (0 '***' ,0.001 '**' ,0.01 '*') are displayed by dark, medium, and light shading of blue colour.

Table S6: The eigenvalue, constrained inertia and cumulative percentage of two first CCA.

Table S7: The principal coordination of canonical metabolic variables of two first axis of CCA analysis. The highest and the lowest values are displayed in green and red colors respectively.

Table S8: Principal coordination of canonical geographical variables of two first of CCA. The highest and the lowest values are displayed in green and red colors, respectively.

Figure S1: The association among measured variables and two first axis of PCA. The first and second dims explained up 20.9% and 12.7% of the total variation respectively. The colors of vectors show the contribution rate of each variable to Dim1 and Dim2.

Figure S2: Dendrogram of the hierarchical clustering analysis based on HCPC

Figure S3. CCA plot of the relationship between morphometric characteristics, environmental, and metabolic variables.

Figure S4: CCA ordination biplot of 50 geographical location of *Trigonella foenume* L.

Excel S1: The average of all measured morphometric characteristics and metabolic traits.

Excel S2: The average of traits in clusters I, II and II based on HCPC analysis.

Excel S3: The average of traits in top five related regions to the first two CCA.

Table S1: List of *Trigonella foenume* L. include their locations, longitudes, and latitudes at 50 regions.

Location	Longitude	Latitude	Location	Longitude	Latitude
Mahnian	49.085535	35.52108	Azna	48.680525	33.488492
Kakareza	48.261338	33.720642	Saveh	50.331931	35.037995
Kuhdasht	47.681523	33.563479	Sabzevar	57.65865	36.224587
Khomein	50.317967	33.663119	Parand	50.987826	35.478324
Mardabad	50.537596	35.693079	Chitgar	51.212207	35.729605
Bandarabbas	56.285105	27.214064	Geshlagh	36.01861	50.419564
Hamadan	48.527574	34.835419	Boyinzahra	35.753171	50.058024
SagzAbad	49.934878	35.766543	Birjand	59.254591	32.862695
Bishe	48.687335	33.444838	Ghadim abad	49.968588	36.218293
Alashtar	48.268144	33.916449	Mahalat	50.461191	33.91693
Borujerd	48.813873	33.805145	Gayene	59.169619	33.565837
Shiraz	52.233507	29.62115	Shushtar	48.832836	32.121287
Khorramabad	47.996006	33.510018	Eslamshahr	51.246368	35.535401
Kazerun	51.944223	29.568908	Aligudarz	49.699415	33.387433
Haji-abad	56.363404	27.25745	BajiAbad	48.630759	31.476271
Minab	56.542827	27.323446	Hamidieh	48.516364	31.435083
Fasa	53.450804	29.137368	MazarSaghi	59.321318	33.145016
Gazvin	49.759621	36.340488	Masjed soleiman	49.188288	32.053383
Joghtay	57.058914	36.618904	Takestan	49.714336	36.078885
Salafchegan	50.472353	34.493022	Mirash	50.731482	36.182436
Kalateh	47.047711	33.760414	Darvan	51.036232	36.006212
Vardij	51.173727	35.810671	Malayer	48.789939	34.294051
Ferdows	58.254167	34.311944	Shazand	49.400067	33.949173
Kashmar	58.485833	35.464167	Nazarabad	50.621609	35.970873
Varish	51.173651	35.829366	Dizan	50.955418	36.224762

Table S2: Equation of calibration curve and R2 value of measured metabolic traits by HPLC and spectrophotometer.

Metabolites	Equation	R^2 value
Aspartic acid	y = 4717.4X - 119157	$R^2 = 0.99$
Glutamic	y = 1581X - 124	$R^2 = 0.99$
Isoleucine	y = 2744x - 2727.9	$R^2 = 0.99$
Leucine	y = 1087.3X - 1851.2	$R^2 = 0.99$
Phenylalanine	y = 1777.1X + 9045	$R^2 = 0.99$
Valine	y = 4998.2X + 2344	$R^2 = 0.99$
Lysine	y = 12375X - 1057	$R^2 = 0.99$
4-hydroxy isoleucine	y = 4108X - 291.2	$R^2 = 0.99$
Trigonelline	y = 455.25X - 200.81	$R^2 = 0.99$
Phenol	y = 0.009X - 0.029	$R^2 = 0.99$
Flavonoid	y = 0.008X - 0.002	$R^2 = 0.99$

Table S3. Correlation matrix between 19 morphometric ant metabolic variables in fenugreek using Pearson's correlation coefficients (r) for cluster I. Significance P-values: *P-value* (0 '***' ,0.001 '**' ,0.01 '*') are displayed by dark, medium, and light shading of blue colour.

Cluster I

	trigonelline	*4-hydroxy isoleucine*	*aspartic acid*	*Glutamic*	*valine*	*phenylalanine*	*isoleucine*	*lysine*	*leucine*	*antioxidant*	*flavonoid*	*Phenol*	*seed weight*	*pod number*	*seed per pod*	*length pod*	*pod width*	*seed length*
4-hydroxy isoleucine	0.12																	
aspartic acid	-0.02	0.24																
glutamic	-0.10	-0.35	0.34															
valine	-0.26	-0.02	0.18	-0.33														
phenylalanine	-0.17	0.04	0.13	-0.39	0.90													
isoleucine	0.36	0.62	0.40	-0.33	0.23	0.15												
lysine	-0.57	0.37	0.07	-0.15	0.23	0.32	-0.17											
leucine	-0.33	-0.40	0.12	0.21	0.66	0.64	-0.35	0.15										
antioxidant	0.13	-0.29	0.08	-0.07	-0.14	-0.09	-0.03	-0.27	-0.39									
flavonoid	0.28	0.08	0.51	0.39	-0.25	-0.11	-0.06	-0.05	0.04	0.16								
phenol	0.12	-0.06	0.17	-0.24	0.23	0.23	0.04	-0.22	-0.08	0.63	0.28							
seed weight	0.70	0.14	0.18	-0.25	0.02	0.06	0.53	-0.43	-0.23	0.33	0.18	0.27						
pod number	0.02	-0.05	0.32	0.29	-0.25	-0.19	-0.02	-0.11	0.01	0.00	0.41	-0.20	0.11					
seed per pod	-0.43	0.31	0.06	-0.19	0.01	-0.04	0.05	0.33	-0.16	0.19	-0.14	0.18	-0.04	-0.38				
length pod	0-39	0.32	0.18	-0.09	0.09	-0.16	0.18	0.19	-0.02	-0.21	-0.05	0.07	-0.19	-0.15	0.65			
pod width	0.24	0.04	0.30	0.12	0.14	0.23	0.04	0.15	0.26	-0.08	0.32	0.01	0.37	0.54	-0.28	-0.11		
seed length	-0.24	-0.02	0.28	0.19	0.13	0.00	-0.04	0.30	0.23	-0.41	-0.13	-0.30	-0.26	0.25	-0.17	0.31	0.48	
seed width	0.07	-0.05	-0.04	-0.08	0.01	0.14	-0.11	-0.05	0.14	-0.11	0.06	0.03	0.18	0.61	-0.45	-0.30	0.66	0.32

Table S4. Correlation matrix between 19 morphometric ant metabolic variables in fenugreek using Pearson's correlation coefficients (r) for cluster II. Significance P-values: *P-value* (0 '***' ,0.001 '**' ,0.01 '*') are displayed by dark, medium, and light shading of blue colour.

Cluster II

	trigonelline	*4-hydroxy isoleucine*	*aspartic acid*	*glutamic*	*valine*	*phenylalanine*	*isoleucine*	*lysine*	*leucine*	*antioxidant*	*flavonoid*	*phenol*	*seed weight*	*pod number*	*seed per pod*	*length pod*	*pod width*	*seed length*
4-hydroxy isoleucine	0.18																	
aspartic acid	0.02	-0.09																
glutamic	0.28	0.02	0.26															
valine	0.14	0.10	0.23	0.23														
phenylalanine	-0.26	-0.14	-0.16	-0.26	0.01													
isoleucine	0.11	0.14	-0.43	0.03	0.30	0.23												
lysine	-0.13	0.31	-0.08	-0.11	0.27	0.06	0.02											
leucine	-0.31	-0.59	-0.10	-0.27	-0.49	0.00	-0.27	-0.12										
antioxidant	0.11	-0.14	0.02	-0.12	-0.31	-0.35	-0.42	-0.32	0.19									
flavonoid	0.14	-0.13	0.13	0.12	-0.39	-0.41	-0.37	-0.43	0.20	0.69								
phenol	0.16	-0.20	-0.15	0.07	-0.50	-0.28	-0.30	-0.56	0.33	0.51	0.79							
seed weight	0.08	-0.05	-0.28	0.16	-0.48	-0.20	0.00	-0.36	0.37	0.23	0.41	0.74						
pod number	0.13	0.03	-0.37	0.03	0.04	0.54	0.40	0.08	-0.10	-0.37	-0.30	-0.02	0.13					
seed per pod	-0.07	-0.01	-0.20	-0.16	-0.38	0.11	0.05	0.14	0.47	0.32	0.27	0.34	0.55	0.17				
length pod	-0.03	0.00	-0.03	0.14	-0.39	0.12	0.10	-0.05	0.33	0.15	0.24	0.27	0.41	0.27	0.75			
pod width	0.27	0.17	0.38	0.36	0.08	-0.19	0.13	-0.03	-0.17	0.19	0.25	0.04	0.06	-0.14	0.38	0.36		
seed length	0.19	0.24	-0.18	-0.03	0.19	0.08	0.14	0.08	-0.40	-0.26	-0.05	0.11	0.11	0.46	-0.05	-0.03	-0.21	
seed width	0.47	0.00	0.11	0.50	-0.06	-0.04	0.39	-0.20	-0.18	0.09	0.27	0.16	0.30	0.34	0.24	0.35	0.60	-0.03

Table S5. Correlation matrix between 19 morphometric and metabolic variables in fenugreek using Pearson's correlation coefficients (r) for cluster III. Significance P-values: *P-value* (0 '***' ,0.001 '**' ,0.01 '*') are displayed by dark, medium, and light shading of blue colour.

Cluster III

	Trigonelline	*4-hydroxy isoleucine*	*aspartic acid*	*glutamic*	*valine*	*phenylalanine*	*isoleucine*	*lysine*	*leucine*	*antioxidant*	*flavonoid*	*phenol*	*seed weight*	*pod number*	*seed per pod*	*length pod*	*pod width*	*seed length*
4-hydroxy isoleucine	-0.02																	
aspartic acid	0.23	0.28																
glutamic	-0.27	-0.51	-0.14															
valine	-0.23	-0.28	-0.48	0.14														
phenylalanine	-0.31	-0.47	-0.38	0.43	0.03													
isoleucine	0.00	0.71	0.07	-0.20	0.11	-0.26												
lysine	0.17	-0.30	-0.26	0.41	0.57	-0.15	0.29											
leucine	0.34	0.09	-0.33	0.08	0.32	-0.12	0.46	0.69										
antioxidant	-0.26	0.36	0.26	-0.08	-0.53	-0.05	0.12	-0.17	-0.22									
flavonoid	0.43	0.33	0.21	-0.58	-0.29	-0.30	0.37	0.13	0.31	0.45								
phenol	-0.15	0.42	-0.26	-0.26	0.17	0.17	0.45	-0.03	0.39	0.09	0.40							
seed weight	0.55	0.11	-0.23	-0.61	0.23	-0.29	-0.02	0.05	0.39	-0.31	0.47	0.47						
pod number	-0.62	0.23	0.01	-0.08	0.24	-0.20	-0.12	-0.34	-0.19	-0.12	-0.47	0.13	-0.07					
seed per pod	-0.04	-0.43	0.14	0.27	-0.30	-0.01	-0.73	-0.37	-0.60	-0.18	-0.62	-0.80	-0.38	0.20				
length pod	-0.02	-0.47	0.19	0.29	-0.55	0.22	-0.61	-0.38	-0.63	0.04	-0.33	-0.70	-0.52	-0.15	0.85			
pod width	0.13	0.38	0.15	0.14	-0.55	-0.26	0.02	-0.25	0.19	0.07	-0.12	0.04	-0.05	0.27	0.25	0.15		
seed length	-0.16	-0.25	0.26	0.55	-0.17	-0.19	-0.30	0.05	-0.37	-0.01	-0.60	-0.84	-0.70	0.13	0.78	0.65	0.26	
seed width	0.36	0.05	-0.01	-0.41	-0.06	0.05	-0.27	-0.29	0.04	-0.01	0.38	0.55	0.78	-0.02	-0.27	-0.32	0.03	-0.68

Table S6: The eigenvalue, constrained inertia and cumulative percentage of two first CCA.

	Two first important axes	
	CCA1	CCA2
Eigenvalue	0.023	0.001
Constrained inertia (%)	0.87	0.07
Cumulative %	0.87	0.94

Table S7: The principal coordination of canonical metabolic variables of two first axis of CCA analysis. The highest and the lowest values are displayed in green and red colors respectively.

Metabolic traits	CCA1	CCA2
Aspartic acid	0.069	-0.212
Glutamic	-0.029	-0.221
4-hydroxy isoleucine	0.374	0.012
Valine	-0.028	0.019
Phenylalanine	-0.111	0.087
Isoleucine	0.190	0.036
Trigonelline	0.046	-0.010
Leucine	-0.064	0.276
Antioxidant	0.027	-0.094
Flavonoid	-0.035	-0.035
Phenol	-0.057	0.050
Lysine	-0.007	-0.002
Pod number	-0.051	-0.003
Seed per pod	-0.120	0.018
Length pod	-0.083	0.014
Seed weight	0.010	0.045
Pod width	-0.042	-0.009
Seed length	-0.026	0.001
Seed width	-0.004	0.045
Solar irradiation	0.564	-0.345
Temperature	0.599	0.294
Rain	0.092	0.963

Table S8: Principal coordination of canonical geographical variables of two first of CCA. The highest and the lowest values are displayed in green and red colors, respectively.

regions	CCA1	CCA2	regions	CCA1	CCA2
Mahnian	-0.968	0.440	Azna	-0.134	-0.405
Kakareza	-1.014	0.388	Saveh	0.112	0.041
Kuhdasht	0.450	0.564	Sabzevar	-0.151	-0.357
Khomein	0.324	1.716	Parand	-0.240	-0.190
Mardabad	-0.706	0.498	Chitgar	-0.060	-0.294
Bandarabbas	1.626	1.872	Geshlagh	-0.650	-0.999
Hamadan	-0.721	0.250	Boyinzahra	-0.650	-0.999
SagzAbad	-1.514	0.224	Birjand	0.662	-1.932
Bishe	-0.127	0.674	Ghadim abad	-0.809	0.092
Alashtar	-0.849	0.027	Mahalat	-1.226	-0.169
Borujerd	-0.287	0.778	Gayene	-0.086	-1.456
Shiraz	-0.586	1.698	Shushtar	1.575	-0.765
Khorramabad	-1.462	0.613	Eslamshahr	0.012	-0.280
Kazerun	1.638	1.765	Aligudarz	0.293	-0.260
Haji-abad	2.241	1.234	BajiAbad	-0.589	0.131
Minab	2.197	1.503	Hamidieh	1.418	-0.669
Fasa	1.933	0.258	MazarSaghi	0.619	-1.720
Gazvin	-0.519	0.842	Masjed soleiman	1.271	-0.572
Joghtay	-0.873	-0.273	Takestan	-0.571	0.720
Salafchegan	-0.332	-0.784	Mirash	-2.275	2.369
Kalateh	0.095	-0.917	Darvan	-0.471	0.491
Vardij	0.003	-0.720	Malayer	-0.692	-0.967
Ferdows	0.235	-1.198	Shazand	-0.424	-0.302
Kashmar	0.926	-1.814	Nazarabad	-0.661	-0.987
Varish	0.037	-0.807	Dizan	-0.463	0.928

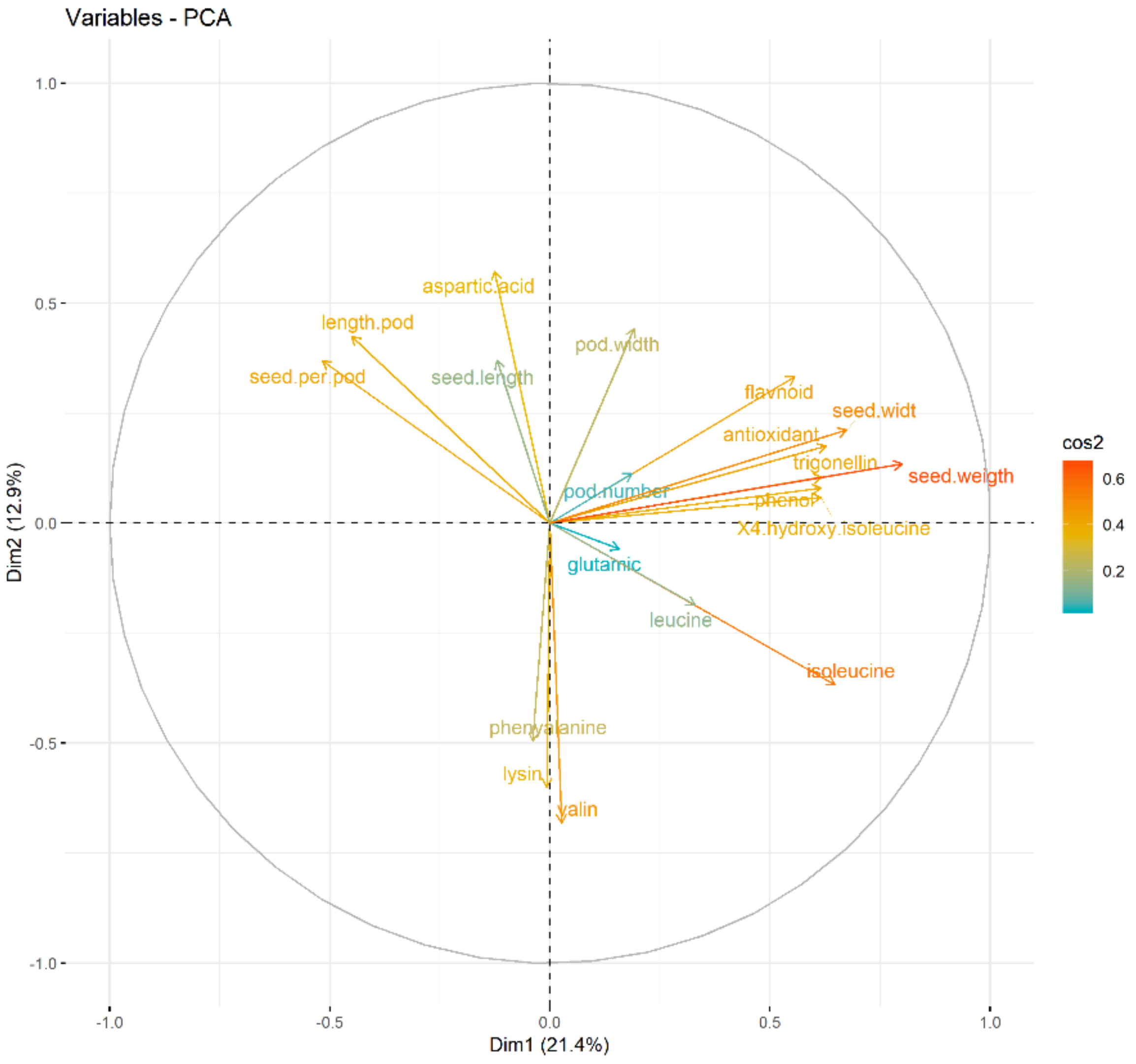

Figure S1: The association among measured variables and two first axis of PCA. The first and second dims explained up 20.9% and 12.7% of the total variation respectively. The colors of vectors show the contribution rate of each variable to Dim1 and Dim2.

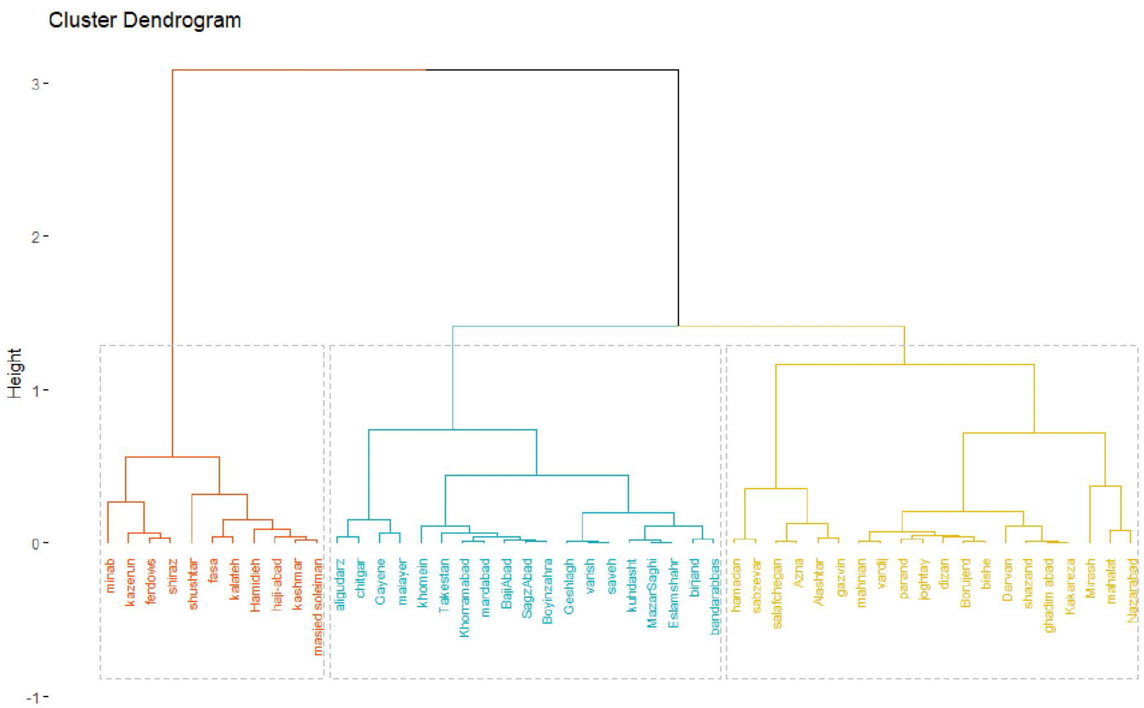

Figure S2: Dendrogram of the hierarchical clustering analysis based on HCPC

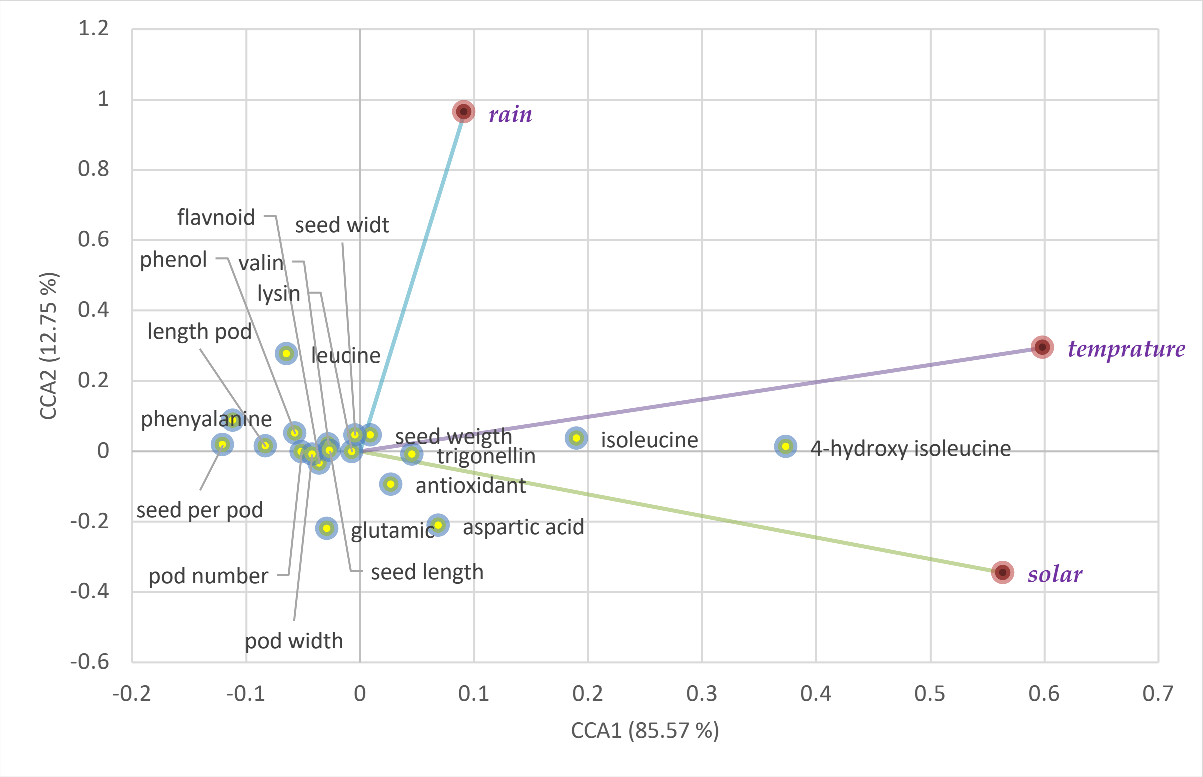

Figure S3: CCA plot of the relationship between morphometric characteristics, environmental, and metabolic variables.

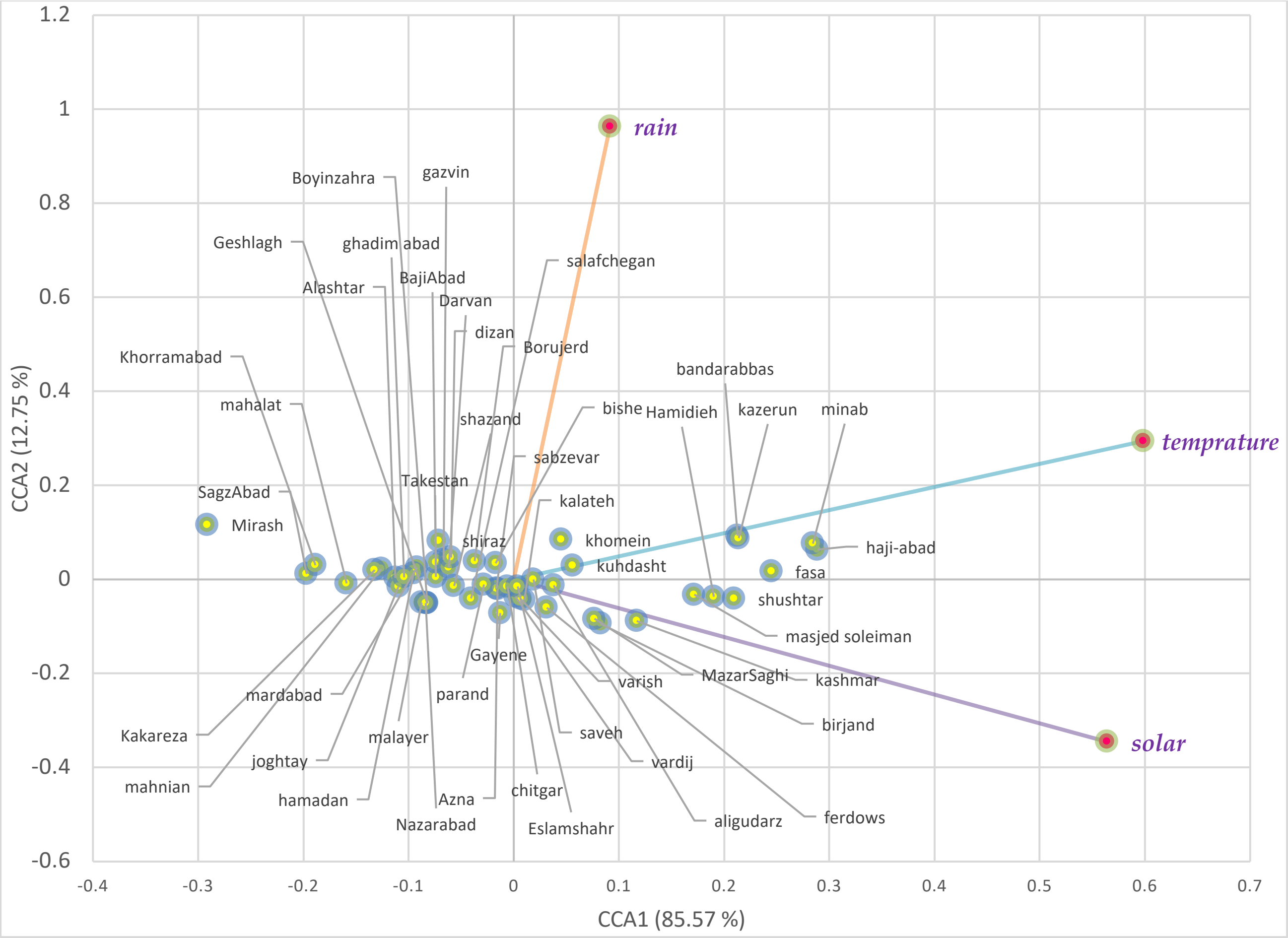

Figure S4: CCA ordination biplot of 50 geographical location of *Trigonella foenume* L.
